# PCL/Graphene Scaffolds for the Osteogenesis Process

**DOI:** 10.3390/bioengineering10030305

**Published:** 2023-02-28

**Authors:** Silvia Anitasari, Ching-Zong Wu, Yung-Kang Shen

**Affiliations:** 1 School of Dentistry, College of Oral Medicine, Taipei Medical University, Taipei 11031, Taiwan; 2 Department of Dental Material and Devices, Dentistry Program, Faculty of Medicine, Universitas Mulawarman, Samarinda 75119, Indonesia; 3 Department Medical Microbiology, Medical Program, Faculty of Medicine, Universitas Mulawarman, Samarinda 75119, Indonesia; 4 Department of Dentistry, Taipei Medical University Hospital, Taipei 11031, Taiwan; 5 Department of Dentistry, Lotung Poh-Ai Hospital, Yilan 265, Taiwan; 6 School of Dental Technology, College of Oral Medicine, Taipei Medical University, Taipei 11031, Taiwan

**Keywords:** PCL, graphene, scaffold, biodegradable, biocompatible

## Abstract

This study aims to characterize the osteoconductivity, optimal bioresorbable, biodegradability, biocompatibility, and mechanical properties of Poly-*ε*-caprolactone (PCL)/graphene (G) scaffolds at concentrations of 0.5, 1, 1.5, 2, 2.5, and 3 wt%, which are used to support bone regeneration through solvent casting and particulate leaching. The water contact angle measurement revealed a transition from a hydrophobic to a hydrophilic surface after incorporating various G concentrations. The scaffolds with 0.5 wt% G had smaller pores compared to those produced using 3 wt% G. Furthermore, numerous pores were connected, particularly those with larger diameters in the 2 and 3 wt% G samples. The proportion of water absorption varied between 50% and 350% for 4 months, with large percentages of scaffolds containing high G concentrations. Raman spectroscopy and X-ray diffraction, which were used to confirm the presence of nanofiller by increasing the ratios of I_D_/I_G_, I_2D_/I_G_, and band 2θ = 26.48°. The mechanical properties were improved by the addition of G, with a Young’s modulus of 3 wt% G, four times that of PCL. Measuring cell biocompatibility, adhesion, proliferation, and differentiation with osteoblast-like (MG-63) cells revealed that PCL/G scaffolds with higher concentrations were more biocompatible than PCL as well as those with lower concentrations.

## 1. Introduction

At the beginning of this decade, natural progenitor cells or autologous cells were considered the best option for regenerating damaged or missing tissue [1]. However, using autologous cells for regenerative purposes can be challenging due to limited tissue volumes, contamination, immune reactions, and difficulty controlling growth and regeneration in 2D cells. To achieve functional integrity, a 3D framework is necessary for complex biological systems. This has led to the integration of cell biology and materials sciences to create degradable biomaterials such as 3D scaffolds made from natural or synthetic polymers which can enhance cell adhesion and proliferation [2].

Several methods and technologies have been developed to produce 3D scaffolds, such as phase separation, self-assembly, electrospinning, emulsion freeze-drying, gas foaming, free radical polymerization, and 3D printing. They allow adherent cells and bioactive molecules to interact with surrounding tissues through the porous structure of the product [3]. For example, synthesized polymeric composite material was fabricated from arabinoxylan (ARX), β-glucan (BG), nano-hydroxyapatite (nHAp), graphene oxide (GO), and acrylic acid (AAc) through free radical polymerization and porous scaffold using the freeze-drying technique. The result found that BGH3 has desirable morphological, structural (with optimum swelling), biodegradation, and mechanical behaviors [4]. Polymeric nanocomposite material was developed using cellulose and a co-dispersed nanosystem (Fe_3_O_4_/GO) by free radical polymerization to fabricate porous polymeric scaffolds via freeze-drying. Antibacterial activities of porous scaffolds were studied against severe Gram-positive and Gram-negative pathogens and increased Fe_3_O_4_ amount in nanosystems with increased antibacterial activities [5]. The synthesis of nanocomposites based on acrylic acid (AAc)/guar gum (GG), nano-hydroxyapatite (HAp NPs), titanium nanoparticles (TiO_2_ NPs), and optimum graphene oxide (GO) amounts via the free radical polymerization method was reported. Increasing the amount of TiO_2_ in combination with optimized GO has improved the physicochemical and microstructural properties, mechanical properties and Young’s modulus, porous properties, and porosity [6]. The combined advantages of PCL and Zn were fused by fabricating PCL/Zn composite scaffolds with different Zn powder contents (1 wt%, 2 wt%, 3 wt%) through deposition modeling. Finally, Zn^2+^ revealed that regulated osteogenesis and osteoclastogenesis by activation of the Wnt/β-catenin and NF-κB signaling pathways, respectively [7]. The polymeric nanocomposite was prepared by free-radical polymerization from sodium alginate, hydroxyapatite, and silica with different GO amounts. The increased GO amount provides different multifunctional materials with different characteristics [8].

This study used a solvent-casting and particulate-leaching method to construct 3D scaffolds which were economical but still showed promising potential to produce porous bone-growth-promoting materials [9,10]. The scaffolds are designed to be biocompatible, biodegradable, and have properties that encourage cell attachment, proliferation, and integration into host tissues for regeneration. These scaffolds also mimic the extracellular matrix (ECM) in a defect area [11,12].

The use of synthetic poly (ε-caprolactone) (PCL), an aliphatic polyester that is biocompatible and biodegradable, has received a lot of attention in bone tissue engineering [2]. However, the lack of mechanical properties of polycaprolactone (PCL) scaffolds restricts their applicability because human cortical and cancellous bones need a higher Young’s modulus. It is, therefore, necessary to combine it with another material, such as graphene (G). Graphene, a two-dimensional (2D) carbon nanofiller with sp^2^-bonded atoms, can be used to improve polymeric materials’ solubility, processing ability, and conductivity. It has a high specific surface area, a poly-aromatic structure, functionalization, and excellent protein adhesion properties [13,14]. Several studies revealed that its concentration affects chemical functionalization through increased hydrophilicity. It also modified the extracellular environment, enhanced osteoblast adhesion and proliferation, and also facilitated differentiation [15].

The combination of PCL and G has been studied as a potential solution to improve the mechanical properties of PCL scaffolds used in bone tissue engineering. Graphene is known to have high mechanical strength and stiffness, which can enhance the Young’s modulus of PCL composites, making them more suitable for use in bones. Therefore, further research is needed to determine the optimal concentration and method of incorporating graphene into PCL to achieve the best mechanical properties [13].

Furthermore, there are concerns about the product’s medical toxicity because it remains in the human body for an extended period as an implantable material. Malhotra et al. [16] have shown that G promoted attachment and proliferation of human neurons, cardiomyocytes, and several types of stem cells without any harmful effects on cell and mitochondrial membranes. Another study by Chang et al. [17] also showed that G promoted bone formation without causing any bone destruction.

Osteoblast-like (MG-63) cells play a crucial role in bone remodeling and bone formation by secreting various proteins such as ECM proteins, cytokines, collagen, and growth factors [18,19]. These cells differentiate into osteocytes for complete bone synthesis and integrate into the bone matrix. The surface properties and toxicity of scaffolds are crucial in promoting osteoblast proliferation at the fracture site, and limited research has been done in this area, especially in relation to waste G and its influence on osteoblast growth [20].

This study focuses on analyzing the impact of different weight percentages of G (0.5, 1, 1.5, 2, 2.5, and 3 wt% G) on the physicochemistry, morphology, mechanics, biodegradation, and biocompatibility of PCL scaffolds. The goal is to identify the scaffolds with the best combination of osteoconductivity, biodegradability, biocompatibility, and physicochemical and mechanical properties to support bone regeneration.

## 2. Materials and Methods

### 2.1. Fabrication of the Scaffolds

**Figure 1:**
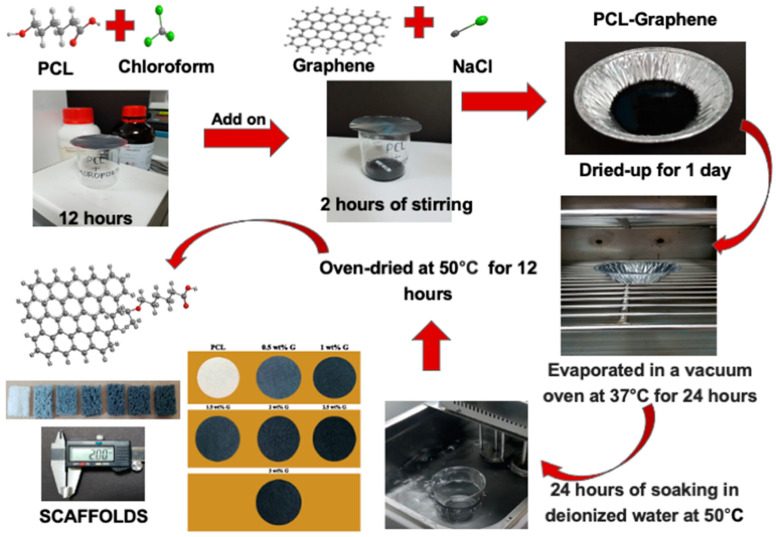
A solvent casting and particulate leaching method for PCL/G scaffold fabrication.

A solvent casting and particle leaching method was used to fabricate PCL and PCL/G scaffolds [10]. PCL (Sigma-Aldrich, Merck, Darmstadt, Germany) was dissolved in chloroform (Honeywell, Charlotte, USA) at room temperature for 12 h. This combination was then mixed with various concentrations of G and NaCl for 2 h. G was previously produced by transferring a graphite intercalation compound into a preheated crucible at 700 °C in a common furnace positioned in the front of a fume cupboard to prevent inhalation of the nanoparticles, and it was left there for 60 s. These layers expanded upon ultrasonication and caused the G to disperse in the solvent. After fabrication, the blend was placed into a cast and cured overnight at room temperature. Chloroform was then evaporated for 24 h at 37 °C in a drying vacuum oven (Deng Yng, Taipei, Taiwan). Deionized (DI) water and a water bath (BH-130D, Taipei, Taiwan) were used to remove porogen from the scaffold. In addition, the DI water was changed every 2 h and then dried in the oven at 50 °C for 12 h. Scaffold fabrication is illustrated in Figure 1.

### 2.2. Characterizations of the Scaffolds

#### 2.2.1. Water Contact Angle (WCA)

The surface property of the PCL/G scaffolds was characterized with a WCA measuring system, which was developed in our laboratory using a sessile drop method. The samples were cut to 10 × 10 mm^2^, and 0.2 μL of a DI water droplets was dropped onto the surface of the scaffold via a motorized syringe at a rate of 1 μL/s. An image was taken at 1 sec, and at least five locations of each PCL/G scaffold were tested, followed by the determination of the average value [2,10].

#### 2.2.2. Water Absorption Rate

Water absorption by the scaffold was evaluated using 1× phosphate-buffered saline (PBS; Gibco-Invitrogen, USA). The samples were immersed in 1× PBS, and their weights were evaluated. Water absorption was calculated using the following equation, where *W*_1_ represents the wet weight and *W*_2_ is the dried weight [21]:

(1) $$Absorption\;rate\;\left(\%\right)=\left(\frac{W_{1}-W_{2}}{W_{2}}\right)\times 100\%$$

#### 2.2.3. Porosity

The porosity of the scaffolds was evaluated by measuring the displacement of ethyl alcohol (EtOH). The initial volume of EtOH was *V*_1_. The total volume of EtOH (Nihon-Shiyaku, Japan) after the scaffold was immersed was *V*_2_. The residual EtOH volume after the scaffold was removed was *V*_3_. The porosity was then calculated using the following equation [13]:

(2) $$Porosity\;\left(\%\right)=\frac{\left(V_{1}-V_{3}\right)}{\left(V_{2}-V_{3}\right)}\times 100\%$$

#### 2.2.4. Pore Sizes

The scaffold morphology and pore sizes were evaluated using scanning electron microscopy (SEM; Hitachi, Japan) at an accelerating voltage of 15 kV. In SEM images, the pores were evaluated using Image-J software. Scale bars that described a known distance were set within the SEM image to measure pore sizes. A pore’s contour was then delineated and calculated (μm). Different cross-sections were passed from the scaffolds [13].

#### 2.2.5. Tensile Test

The tensile strength of the PCL/G scaffolds was determined using a universal testing machine (Shidmazu, Japan) equipped with a 250-N load cell. Experiments were performed at room temperature and a crosshead speed of 3 mm/min. The samples were prepared by cutting a scaffold with a dimension of 40 × 20 × 10 mm^3^. The stress vs. strain graphs for each was used to calculate the Young’s modulus, ultimate tensile strength, and elongation-at-break using the linear region (elastic region) of the graphs. The ultimate tensile strength (*σ_max_*) was calculated using the following equation [22]:

(3) $$\sigma_{max}=P/a$$

where *P* represents the tensile force and *a* is the cross-sectional area.

Young’s modulus (*E*) was determined using the equation [22]:

(4) E=σ/ε

where *σ* represents stress and *ε* represents strain.

Elongation-at-break (𝜀*b*) was calculated using the equation [22]:

(5) εb (%)=∆L/L×100%

where ∆L represents elongation at rupture and *L* represents initial gauge length.

#### 2.2.6. Raman Spectroscopy

PCL/G scaffolds were analyzed using Raman spectroscopy (UniDRON, CL Tech, Taiwan). The samples were folded and mounted on glass slides for measurement with a laser at 457 nm, 50 mW, 1% neutral density filter, 50× objective lens, 1 s exposure length, 60 s average time, and a signal normalization at a peak of 2918 cm^−1^ for processing. Origin Pro 2022 software was used to analyze the data, which ranged from 500 to 3300 cm^−1^ [23].

#### 2.2.7. X-ray Diffractometer (XRD)

The XRD spectra for PCL/G scaffolds were produced on a high-power (18 kW) XRD (Rigaku, TTRAX3, Japan). The determinations were carried out using radiation of λ = 1.54 Å in a range of 2θ = 10~50° at a scan rate of 4°/min. They were then analyzed by fitting a Lorentzian curve for height (intensity) using Origin Pro 2022 software [23].

### 2.3. Biodegradation Time Test

Biodegradation of the PCL/G scaffolds with a dimension of 10 × 10 × 2 mm^3^ was determined by placing them in a tube containing 5 mL of 1× PBS (Gibco-Invitrogen). The samples were then sealed with parafilm and placed in a water bath at 37 °C for 4 months without refreshing the 1× PBS. Every month, the scaffolds were removed from the water bath, rinsed five times with DI water, and dried for at least 24 h in a vacuum dryer. Raman spectroscopy and XRD were used to examine the samples [10].

### 2.4. In Vitro Cell Culture

#### 2.4.1. Scaffold Preparation and Cell Seeding

Scaffolds used for cell culture had a dimension of 10 × 10 × 2 mm^3^ and contained various G weight ratios. They were sterilized in a 95% ethanol solution for 24 h, followed by washing in a 1× PBS solution three times to eliminate residual ethanol. Before cell seeding, scaffolds were incubated for 3 h in Dulbecco’s modified Eagle medium (DMEM; Gibco-Invitrogen).

Osteoblast-like (MG-63) cells at passage 5 (kindly provided by 3D Global Biotech Inc, Taipei, Taiwan) were cultured in culture plates with DMEM containing 10% fetal bovine serum (FBS) and 1% penicillin in an incubator at 37 °C with 5% CO_2_. The medium was replaced every 2–3 days, and they were digested and subcultured using 0.25% of trypsin-EDTA (Gibco, USA) for detachment after 80% confluence was achieved [13,21].

#### 2.4.2. MTT Assay (3-(4,5-Dimethylthiazol-2-yl)-2-5-diphenyltetrazolium bromide)

MTT (a tetrazole) assay was used to examine the biocompatibility and proliferation of osteoblast-like (MG-63) cells [8].

##### Biocompatibility

The surface area of each scaffold was measured with following formula [24]:

(6) Total Surface Area=2πrh×2π 

where *π* is 3.14, *r* is the radius, and *h* is the height.

Subsequently, DMEM supplemented with 10% FBS and 1% of penicillin/streptomycin was added with the formula:

(7) $$Total\;medium\;\left(\mathrm{mL}\right)=\left(Total\;Surface\;Area\right)/6$$

The scaffold and DMEM were placed in a 50 mL conical centrifuge tube and shaken in a shaking water bath at 37 °C and 100 rpm for 24 h. The extracts were filtered with a Millipore filter unit (Sartorius, France) with a pore size of 0.22 μm and a polyethersulfone (PES) membrane.

Osteoblast-like (MG-63) cells were detached using 1% trypsin-EDTA, and 100 μL of a cell suspension at a concentration of 10^5^ cells/mL was seeded into a 96-well plate. Furthermore, the plates were placed in an incubator at 37 °C with 5% CO_2_ for 24 h. The medium was then removed and replaced with extracted samples, which were incubated for another 24 h. An MTT-labeling agent reagent of 50 μL was added to each well and then placed in an incubator at 37 °C with 5% CO_2_ for 3–4 h. The reagent was then removed and solubilization buffer was added to each well to dissolve the purple formazan crystals. Optical density was measured at 570 nm using an enzyme-linked immunosorbent assay (ELISA) reader. The optical density of cells was obtained to determine the cell biocompatibility using the following equation [13,24]:

(8) $$\mathrm{Cell}\;\mathrm{biocompatibility}\;\left(\%\right)=\frac{\mathrm{OD}\;\mathrm{sample}}{\mathrm{OD}\;\mathrm{control}}\times 100\%$$

##### Proliferation

Cells were detached using 0.25% trypsin-EDTA (Gibco-Invitrogen), and each sample was seeded with 0.5 mL at a concentration of 10^4^ cells/mL in 24-well plates, which were placed in an incubator for 21 days. The medium was renewed every 2–3 days during this period. Furthermore, the cells were removed from the culture incubator to evaluate the results on days 1, 4, 7, 14, and 21. A total of 50 μL of MTT-labeling reagent was then added to each well. After 4 h of incubation at 37 °C, the reagent was removed, followed by the addition of a solubilization buffer. The absorbance at 570 nm was determined to establish cell proliferation [2,13].

#### 2.4.3. Alkaline Phosphatase (ALP) Assay (Differentiation Assay)

A commercial ALP test kit was used to detect ALP activity (AnaSpec, Fremont, CA, USA). An ALP dilution buffer was prepared by diluting 10× to 1× assay buffer using DI water. The alkaline phosphatase standard of 10 μg/mL was then diluted to 0.2 μg/mL using the dilution buffer. The ALP standard solution was serially diluted by two-fold to yield concentrations of 0, 3.1, 6.2, 12.5, 25, 50, and 100 ng/mL. The wells were filled with 50 μL of solutions ranging 0–200 ng/mL. The samples were cultivated for 21 days, and they were removed from incubator to evaluate on days 1, 4, 7, and 21. Samples were washed twice with 1× assay buffer upon removal from the incubator. The extract buffer (200 μL; 10 mL 1× assay buffer plus 20 μL Triton X-100) was then added to each well for cell extraction. The samples were held at 4 °C for 10 min under agitation. Cell suspensions were then transferred to 1.5 mL tubes and centrifuged for 10 min at 4 °C and 2500× *g*. A total of 50 μL of supernatant was transferred to a 96-well plate for each sample. Subsequently, 50 μL of a pNPP substrate solution as well as ALP standard were added to each well, followed by incubation for 30 min at the desired temperature. The 96-well plate was shielded from light throughout this process, and the reaction was stopped by the addition of 50 μL of stop solution. The absorbance at 405 nm was then determined using an ELISA reader [12,21].

#### 2.4.4. Cell Morphology and Adhesion

Cell adhesion at the surface of the scaffold was evaluated by scanning electron microscopy (SEM). The samples were washed with PBS after the medium was removed, followed by fixation with 0.6 mL of 2.5% glutaraldehyde in a PBS solution for 30 min at 4 °C. After being washed twice with PBS, the scaffolds were dehydrated in ethanol of 30%, 50%, 70%, 90%, and 100% and then dried in HMDS. Subsequently, they were gold-coated using a sputter coater and viewed with SEM at an accelerating voltage of 5 kV [13,21].

### 2.5. Statistical Analysis

All experimental data are presented as the mean ± standard error (SE) for each group of samples. All experiments had at least three scientific replicates. The data obtained were analyzed using SAS software. A one-way analysis of variance (ANOVA) and Tukey’s post hoc test were used to determine relevant differences in data. However, if the distribution was not normal and homogeneous, it was analyzed using the Kruskal–Wallis’s test and Mann–Whitney significant difference post hoc test to assess the differences between groups. Significance levels were set at * *p* < 0.05, ** *p* < 0.01, *** *p* < 0.001, and **** *p* < 0.0001 [19,21].

## 3. Results and Discussion

Several studies revealed that the surface properties of a scaffold are some of the most important qualities which determine cell adherence. On hydrophobic surfaces, a dense layer of non-specific proteins can displace water from the surface and instantly aggregate on the materials. Meanwhile, a hydrophilic surface allows the attachment of chemicals that improve adhesion. These properties are influenced by low-stiffness and high-stiffness scaffolds [25].

**Figure 2:**
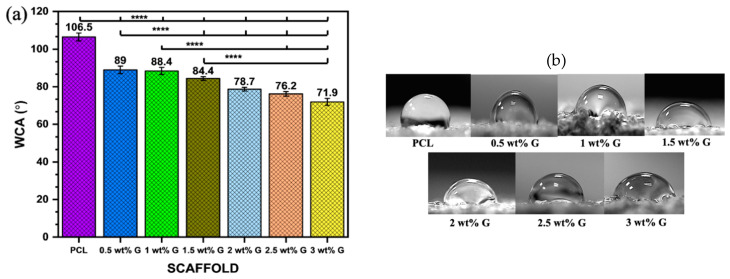

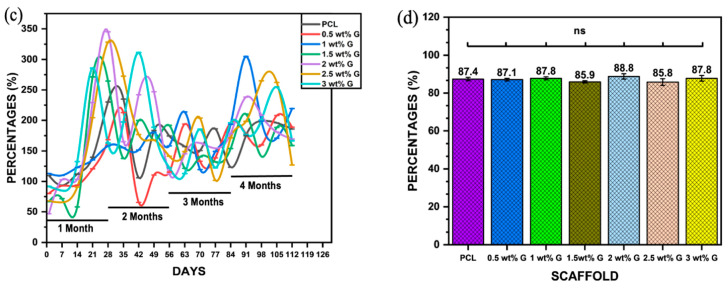
Physical properties: (**a**) water contact angle of PCL/G scaffold; n = 6; **** *p* < 0.0001. (**b**) Photograph of WCA of scaffold. (**c**) Water absorption rate of PCL/G scaffold; n = 3. (**d**) Porosity of PCL/G scaffold n = 9.

WCA was examined on the solid surfaces of PCL and PCL/G scaffolds with various G concentrations to determine the effects of different concentrations on the wettability of the samples. When a liquid drop makes contact with a solid surface, it either retains its drop-like shape or spreads out on the solid surface, and this property is characterized by using water contact angle (WCA) measurements [26]. The liquid droplet tends to form an angle with the solid surface when it is placed in contact with it as shown in Figure 2a,b. The results showed that the WCA decreased as the proportion of G increased from 106.5° ± 2.1 in PCL to 71.9° ± 1.9 at 3 wt% G (*p* < 0.0001). This indicated that the hydrophobicity of PCL/G scaffolds was marginally reduced due to its addition. The reduced hydrophobicity is attributed to the wrinkled surface of graphene, which has a hydrophilic chemical composition [27].

The studies by Al-Azzam et al. [28] and Zhang et al. [29] reported that mostly mammalian cells adhere best to moderately hydrophilic surfaces with a WCA between 40 and 75°. An increase in hydrophilicity leads to an increase in protein adsorption and reduces scaffold toxicity, which plays a crucial role in cell attachment. The interaction between cells and components of the extracellular matrix (ECM) such as fibronectin, vitronectin, collagen, and laminin can impact cell attachment and migration, as shown in Figure 2b. This study revealed that the addition of 3 wt% G to scaffolds also continuously improved cell proliferation compared to PCL due to its hydrophilic surface. 

However, superhydrophilic (WCA < 5°) and superhydrophobic (WCA > 150°) surfaces can hinder cellular attachment and spread due to weak binding of cell-adhesion-mediating molecules. This weak binding causes cells to dissociate when multiple cells interact with the surface simultaneously, leading to limited or prevented cellular adherence and spread. [28,30].

Another physical characteristic that must be determined is the water absorption rate, which is essential for evaluating a composite material’s suitability for bone tissue regeneration. This is because it represents the effectiveness of body fluid absorption and nutrient transfer [18,31]. Figure 2c shows the water absorption rates of the PCL and the PCL with G (a hydrophilic material) over a 4-month period in PBS solution. The results showed that samples containing G had higher water absorption than PCL due to the hydrophilic properties of G. The percentage of water absorption varied from 50% to 350% during the 4 months, with the highest values observed in samples containing 2, 2.5, 1.5, and 3 wt% G in the first month, but only 2 and 3 wt% G maintained a high volume of PBS throughout the second month. By the third month, every scaffold’s capacity had been reduced, although the capacities of 1, 2, 2.5, and 3 wt% G increased yet again in the fourth month. The results suggest that the water absorption capacity can be improved by controlling the WCA, porosity, and pore size of the scaffold [31,32].

Apart from the WCA and water absorption rate ability, other physical properties support the production of a suitable scaffold. The porosity and pore size on the surface and the interior are required for cell distribution and placement. They are also needed for the exchange of nutrients, gases, and metabolic by-products between the exterior environment and the interior of the scaffold [29,31]. In this study, there was no statistically significant difference (*p* > 0.05) in the porosity of PCL compared to PCL/G at various concentrations. The values obtained ranged from 85.8 ± 1.85% to 88.8 ± 1.4%, as shown in Figure 2d. This showed that the porosity of the scaffold was more comparable to that of trabecular bone (50–90%) compared to cortical bone (5–15%) [32].

Porosity needs to be increased in the scaffold’s surface and within its area, which can enhance the rate of water uptake. This condition can alter the level of fluid shear on cells, thereby causing adherence and proliferation on the scaffold. However, there is restriction of cellular movement as well as interchange of nutrients and metabolic waste if the pores are not interconnected. The solvent casting and particulate leaching were promising methods according to Lutzweiler et al. [33]. The size and interconnection of pores could be controlled based on the size of the salt as a porogen. Additionally, the high porosity of the scaffold (>85%) could also control the interconnected pores [34].

**Figure 3:**
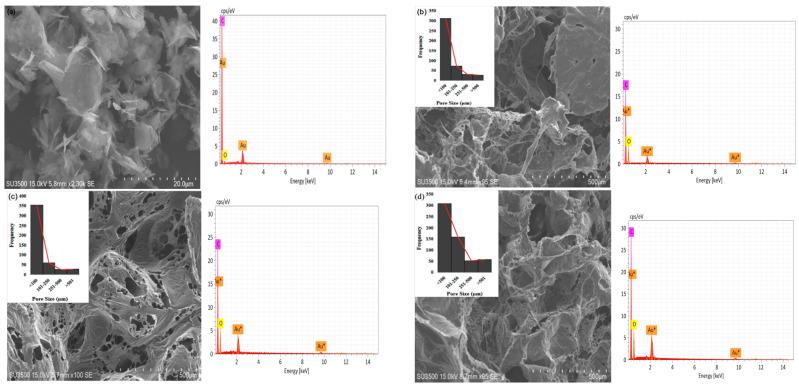

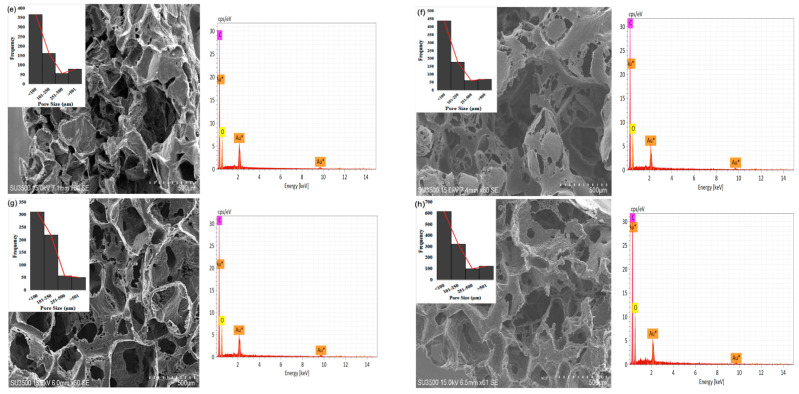
SEM of morphology, pore distribution, and SEM-EDS of scaffold: (**a**) graphene; (**b**) PCL; (**c**) 0.5 wt% G; (**d**) 1 wt% G; (**e**) 1.5 wt% G; (**f**) 2 wt% G; (**g**) 2.5 wt% G; (**h**) 3 wt% G.

The study showed that the 3 wt% G sample has a greater number of pores with diameters of <100 μm (616), >101 μm (548), and >501 μm (124) compared to the others, as shown in Figure 3a–g. The 0.5 wt% G had three times more macropores with a size of <100 μm compared to >101 μm, while PCL had 2.5 times more macropores of size <100 μm, as shown in Figure 3b,c.

As osteoblasts ranged from 10 to 50 μm and fibroblasts ranged from 10 to 15 μm, the pore size of the scaffold must be <100 μm for fibroblast ingrowth, while >100 μm is suitable for osteoblast proliferation. This indicates that a PCL/G scaffold with a high concentration (2, 2.5, and 3 wt%) of G is appropriate for osteoblast ingrowth, as shown in Figure 3f–h. Several studies revealed that micropores of 10 μm were important for enhancing osteoinduction. This was because they were related to the formation of non-mineralized osteoid or fibrous tissues, which can increase the number of cytokines produced by fibroblasts. Furthermore, fibroblasts can increase osteoclast multiplication, inhibit osteoblast functions, and induce local inflammation [35,36].

Vascularization is another component that influences osteogenesis. Wang et al. [37] showed that the use of scaffolds with pore sizes of 525 μm increased osteogenesis and vascularization due to newly formed arteries providing appropriate oxygen and nutrients for osteoblastic activity within the larger pores of the scaffolds. This led to osteopontin (OPN) upregulation, chondrogenesis (collagen type I), and bone mass production. Additionally, graphene materials have excellent angiogenesis properties, which is important for osteogenesis [38] because poor vascularity can hinder the regeneration of complex tissues such as bone [37,39].

**Figure 4:**
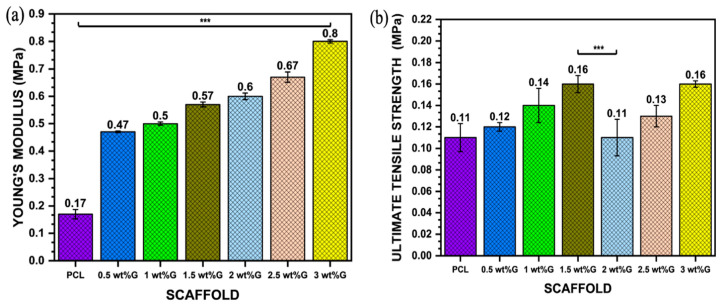

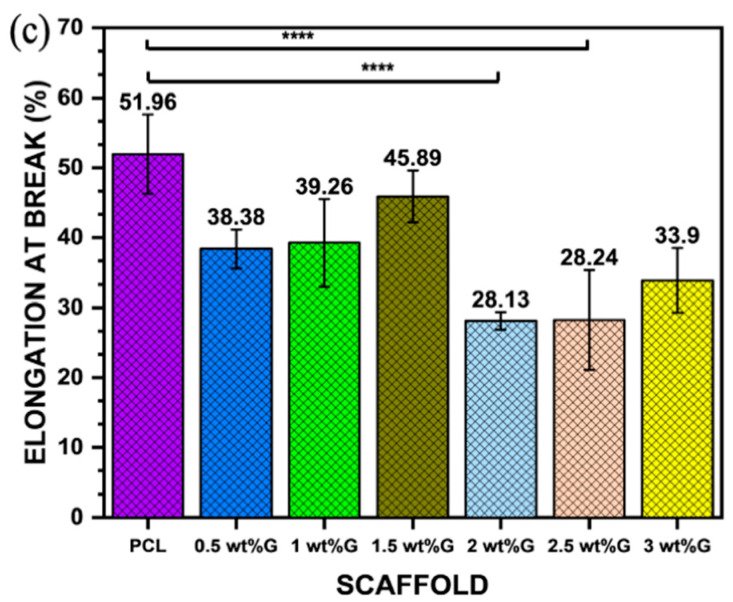
Mechanical properties: (**a**) Young’s modulus value of PCL/G scaffold; n = 3; *** *p* < 0.001. (**b**) Ultimate tensile strength (*σ_max_*) of PCL/G scaffold; n = 3; *** *p* < 0.001. (**c**) Elongation-at-break (*ε_b_*) of PCL/G scaffold; n = 3; **** *p* < 0.0001.

The mechanical properties of 3D scaffolds are an important design factor because of their impact on biostability. PCL has strong covalent bonds but weak van der Waals bonds, resulting in lower strength. However, incorporating graphene into PCL can increase strength due to the alignment of large molecules and decrease the influence of weak van der Waals bonding. This is why PCL/G composites with high graphene content have good strength and stiffness (Young’s modulus) despite having larger pore sizes than PCL, as shown in Figure 4a,b (*p* < 0.001) [40,41]. Furthermore, the mechanical properties of the scaffold, such as its ultimate tensile strength and Young’s modulus, play a role in regulating osteoblast behavior by affecting cell–ECM interactions. This interaction between the scaffold, ECM, and cells creates a complex microenvironment that influences cell behavior through mechanosensing. It enhances the ability of the cells to generate traction forces and enter the cell cycle, resulting in increased spreading and proliferation [40,42].

The addition of G to polymer materials increases the ultimate tensile strength of the material but reduces its ductility. This is shown in this study, where the addition of G to PCL in a sample with 3 wt% G resulted in an increase in ultimate tensile strength (*p* < 0.001) but a reduction in elongation-at-break (𝜀*b*) (*p* < 0.0001), which is related to the strain of the substrate as shown in Figure 4b,c. Moreover, the tensile strain of the substrate promoted osteoblast ECM formation by increasing integrin density on the surface of the ECM, such as integrin1 mediating osteoblast differentiation [43,44].

**Figure 5:**
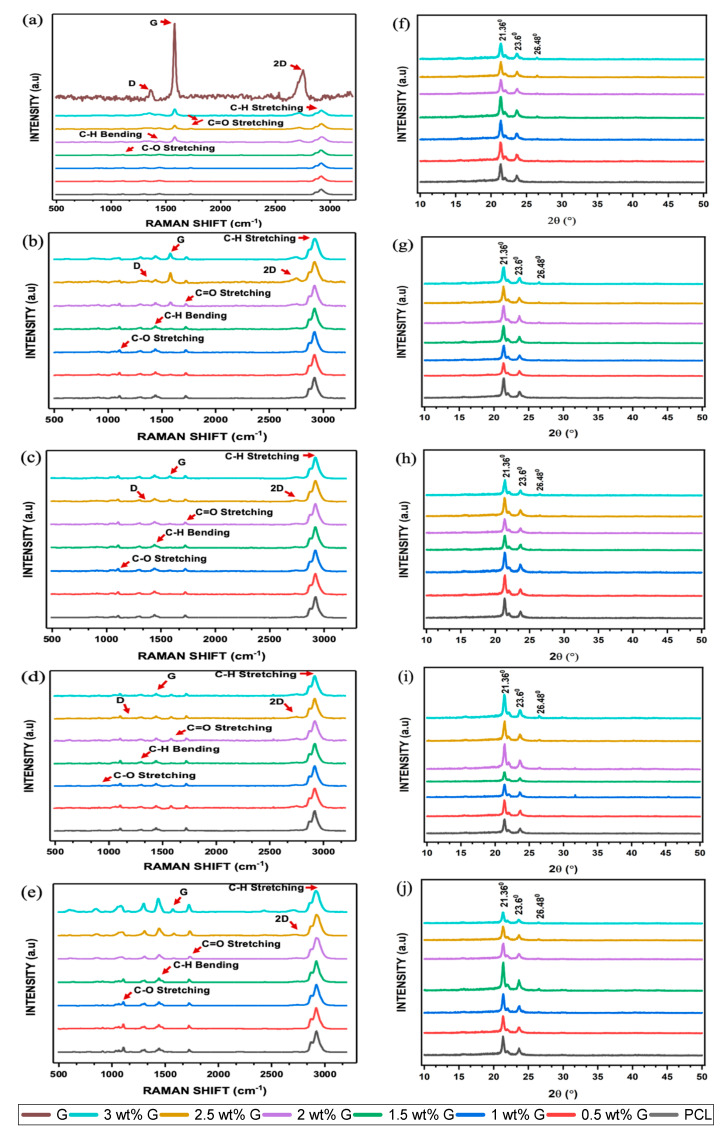
Raman spectroscopy of PCL/G scaffold. (**a**) Initial; biodegradation process: (**b**) 1 month; (**c**) 2 months; (**d**) 3 months; (**e**) 4 months. X-ray diffraction of PCL/G scaffold. (**f**) Initial; biodegradation process: (**g**) 1 month; (**h**) 2 months; (**i**) 3 months; (**j**) 4 months.

Raman spectroscopy and X-ray diffraction methods are relatively accurate at determining the chemical structure of various materials. Furthermore, Raman spectroscopy can also detect changes in vibrational spectral features which are induced by the production of defects, crystal disorder, edge structures, oxidation, or changes in the number of layers of the high activity. These changes can occur because of certain factors. On the spectrum, G displayed all four properties, namely D, G, D’, and 2D bands at 1320–1350 cm^−1^, 1580–1605 cm^−1^, 1602–1625 cm^−1^, and 2640–2680 cm^−1^, respectively. The presence of disorder in the aromatic structure or the edge effect of G due to oxidation is associated with the D peak, while the G peak was caused by the stretching of C-C bonds. The 2D peak is related to the thickness and can also be used to identify the number of layers as well as the quality of the aromatic rings [44,45]. The addition of G caused an increase the peaks of the D, G, and 2D bands, and this was clearly evident in the 2, 2.5, and 3 wt% G scaffolds, as shown in Figure 5a.

The intensity ratio of the D to G bands, also known as I_D_/I_G_, is a measurement that can be used to determine the level of disorder or covalent bond. In this study, the I_D_/I_G_ showed a slight increase as the concentration of G increased, as shown in Table 1. An increment in this ratio indicated the successful covalent bonding of G to oxygenous groups [46], which led to the introduction of a significant number of defects. A covalent bond happened between free radicals (salt) and C=C bonds of graphene. When salt was heated, a highly reactive free radical was produced, which attacked the graphene sp^2^ carbon atoms, forming a covalent bond, and the degree of a covalent functionalization reaction was shown in the ratio of I_D_/I_G_ [47]. Furthermore, defects in the scaffold are responsible for an increased oxygen content, as shown in Figure 3a–h, which causes a reduction in its toxicity and increases cell adhesion [48,49]. The higher the number of oxygen-containing functional groups on the surface of a material, the better its hydrophilic qualities, and this has a significant effect in enhancing cell viability. The I_2D_/I_G_ ratio of PCL/G showed a slight increase in the 2 wt% G to 3 wt% G, which indicated an increasing number of G layers [Table 1]. Previous studies revealed that the number of layers is an important parameter due to its ability to increase the surface area and the bending stiffness [49,50].

The results of XRD experiment are in line with that of the Raman spectroscopic analysis. Two major peaks were found at 2θ = 21.36° and 23.6° in the diffraction pattern of the semicrystalline PCL. Furthermore, the addition of G did not have a substantial impact on 2θ = 21.36°, but there was a slight decrease at 2θ = 23.6°, as shown in Figure 5f. The peak at 2θ = 26.48° improved as the concentration of G increased. Previous studies showed that increasing its concentration led to an increment in functionalized oxygen. It also enhanced the capacity of G to disperse in water or cell culture media, which can increase cell viability [51,52].

The biodegradation of scaffolds is an important factor to consider when analyzing their biological characteristics. This parameter was explored at a duration of 4 months by submerging the samples in 1× PBS at 37 °C. Biodegradation was then assessed using Raman spectroscopy and XRD to determine its progression. PCL is a polyester containing ester groups (C=O-O) and cyclic alkyl groups. The pre- and post-biodegradation PCL spectra had three significant absorption peaks, which are presented in Figure 5b–e. Absorption bands located around 2900 and 2800 cm^−1^ were attributed to asymmetric and symmetric C-H stretching, those located between 1730 and 1750 cm^−1^ were assigned to C=O stretching, and the band located at 1150 cm^−1^ was linked to the presence of C-O stretching. After biodegradation, the intensity of PCL in the spectrum decreased, and this confirmed the occurrence of the process. The highest intensity of the change in asymmetric and symmetric C-H stretching occurred at 3 months, while those of C=O and C-O stretching were observed at 4 months. The ability of the scaffolds to absorb water decreased due to the absence of these peaks, which are capable of forming hydrogen bonds with water molecules [53,54].

I_D_/I_G_ was analyzed as part of the G biodegradation evaluation. During the initial phases, the ratio increased due to the addition of G but later decreased. Meanwhile, the intensity of I_2D_/I_G_ increased in the G band. This shows that oxidation continued to cause biodegradation until all D, 2D, and G bands had disappeared, indicating the complete disintegration of G structure, as shown in Table 1 [54,55].

The XRD biodegradation process is illustrated in Figure 5g–j and Table 2. At 1 and 2 months, the peak at 2θ = 21.36° was similar for all scaffolds. However, at 3 months, the peak at 2θ = 21.36° had decreased for the 0.5, 1, and 1.5 wt% G, while it had increased for the 2, 2.5, and 3 wt% G. Comparison of the peaks of 0.5, 1, and 1.5 wt% G to those of 2, 2.5, and 3 wt% G at 2θ = 23.6° are presented in Figure 5g–j. The peak of 2θ = 23.6° in the two groups revealed that their intensities were reduced between 1 and 2 months. The values then increased at 3 months for 2, 2.5, and 3 wt% G scaffolds before decreasing again at 4 months, but the other groups showed the opposite condition. This finding is relatively similar to that of Raman spectroscopy, which showed that the peak associated with the mediated biodegradation process had increased [20,56].

Based on these results, G, when used as a nanofiller, can have a positive influence on the biodegradation rate of PCL and other polyesters because the hydrolytic biodegradation of other aliphatic polyesters was slowed or delayed by non-G materials. It can also have a positive effect on the hydrophobicity of the polymer, which leads to a rapid biodegradation of the PCL [16,20].

The next problem is the waste products caused by the biodegradation of the scaffold. Several studies have reported the ability of G biodegradation product to biodegrade or biotransform into less-reactive forms as well as to be naturally eliminated from the body [56,57]. Lasocka et al. [58] stated that scaffolds with the nanofiller generated a considerable increase in average cell mitochondrial activity, which indicates that they are harmless and can promote cell proliferation.

**Figure 6:**
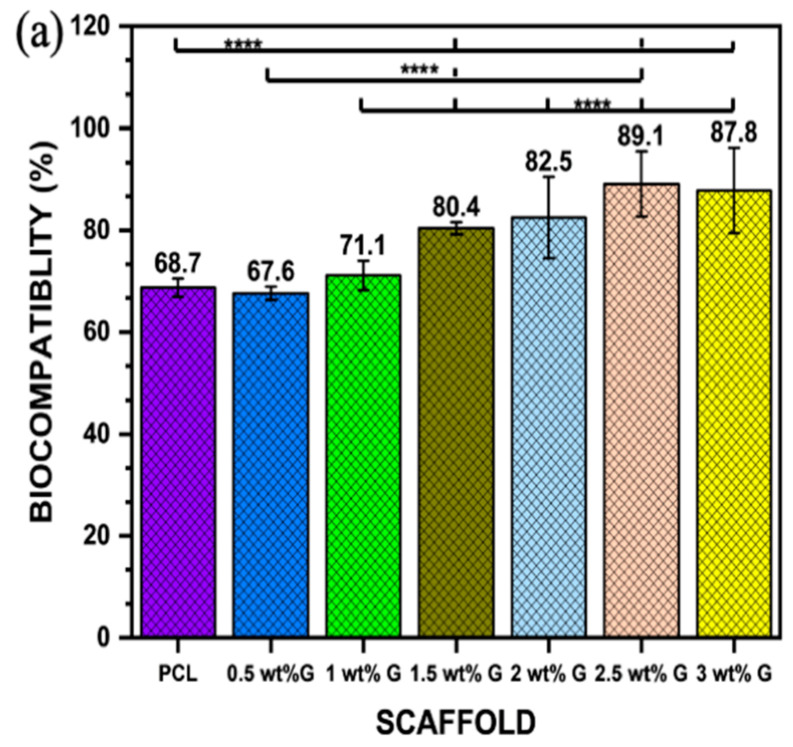

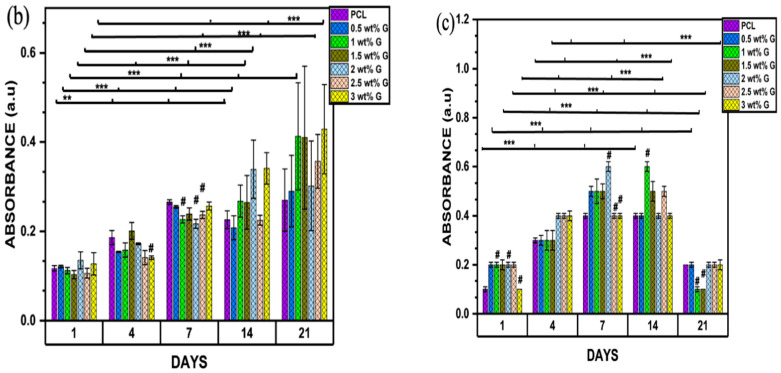
(**a**) Biocompatibility of osteoblast-like (MG-63) cells; n = 6; **** *p* < 0.0001. (**b**) Proliferation of osteoblast-like (MG-63) cells on PCL/G scaffold; n = 6; compared to PCL groups. ** *p* < 0.01, *** *p* < 0.001 (**c**) Alkaline phosphatase of osteoblast-like (MG-63) cells; n = 6; compared to PCL groups. *** *p* < 0.001.

Osteoblast-like (MG-63) cells were cultured for biocompatibility for 24 h, followed by 21 days of proliferation and differentiation. The respective MTT assay results are presented in Figure 6a. An extract containing 2.5 wt% G was shown to have a higher biocompatibility, followed by 3 wt% G (*p* < 0.0001). However, the values of PCL and 0.5 wt% G were less than 70%, indicating that they were cytotoxic, while the other samples showed values greater than 70%. This indicates that all the scaffolds except PCL and 0.5 wt% G were appropriate for the growth of cells [59].

The MTT assay for cell proliferation showed that the concentrations of 1, 1.5, and 3 wt% G increased steadily from day 1 to day 21, but the value for 3 wt% G was greater compared to the others (*p* < 0.001). This shows that they were suitable for the growth of osteoblast-like (MG-63) cells due to their consistent growth over a period of 21 days. Nevertheless, PCL and 0.5 wt% G increased from day 1 to day 7, decreased on day 14, and then increased slightly on day 21 (*p* < 0.001). This current study revealed that scaffold properties, such as physical (WCA) or mechanical (Young’s modulus) characteristics, have a correlation. They also increase the phase of cell proliferation by prolonging cell growth or inhibiting cellular differentiation, as shown in Figure 6b,c. The MTT result on day 21 increased, while that of the ALP declined [60,61].

ALP acts as a marker of osteoblast differentiation, and its activity in osteoblast-like (MG-63) cells was evaluated on days 1, 4, 7, 14, and 21. PCL and 3 wt% G had lower absorbances than the others on day 1 (*p* < 0.001), as shown in Figure 6c. On day 4, the ALP activities of cells were higher compared to the previous days. The values obtained for 2, 2.5, and 3 wt% G scaffolds were considerably higher than that of PCL and the other PCL/G scaffolds (*p* < 0.001). On day 7, the 1, 1.5, and 2.5 wt% G showed a steady increase which continue to day 14, while 3 wt% G absorbance was constant from day 4 to 14. All the ALP values of the scaffold decreased on day 21, particularly 1 and 1.5 wt% G. When compared to proliferation result, the 1, 1.5, and 3 wt% G samples increased greatly compared to the others on day 21, but the absorbance of 3 wt% G slowly decreased compared to the 1 and 1.5 wt% G, as shown in Figure 6c. Suh et al. [62] stated that the osteoblast proliferation was retarded, while the production of ALP increased. Osteoblast growth showed decreased differentiation activities during the period of rapid proliferation. As the cells slowly proliferated, they began to produce more ALP. The finding showed that the PCL/G scaffold was suitable for osteoblast growth because high concentration of ALP for long duration induced higher frequency of bone fractures (osteomalacia), which led to enlarged or abnormal bone shape due to decreasing bone mineral density [63].

**Figure 7:**
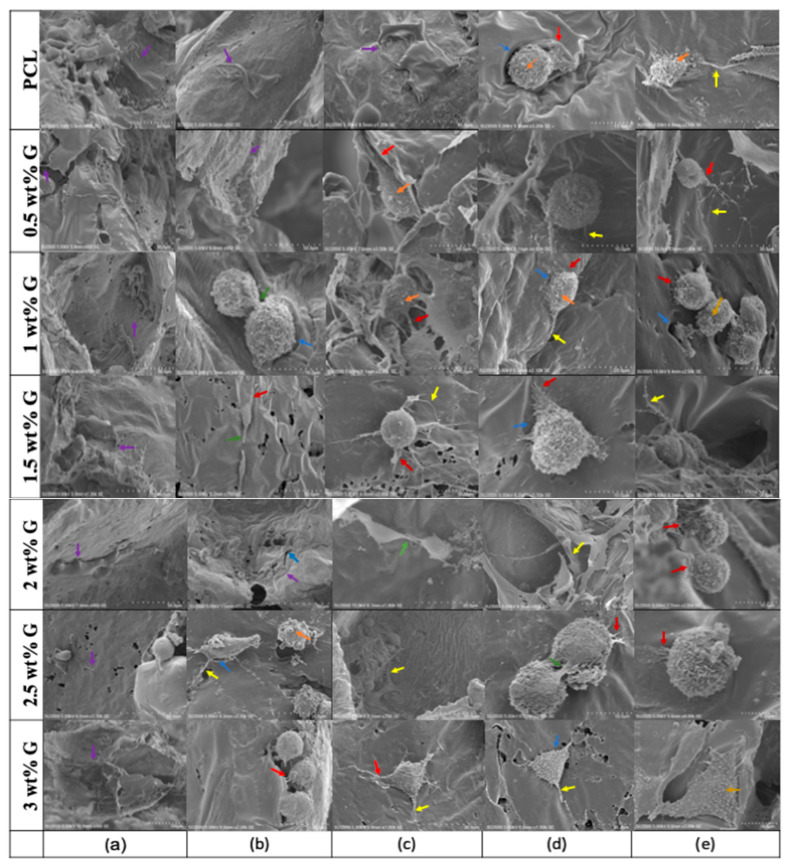
SEM showing the morphology of osteoblast-like (MG-63) cells on days (**a**) 1, (**b**) 4, (**c**) 7, (**d**) 14, and (**e**) 21. Protrusion of cellular body and pores on the surface of scaffold: 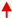 = lamellipodia; 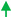 = intercellular connections; 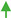 = filopodia and micro-vesicles.

SEM analysis was also carried out on osteoblast-like (MG-63) cells. On the first day (Figure 7a), the cells were uniformly distributed and adhered to the scaffolds at various concentrations. Furthermore, protruding cell membranes were observed on day 4 (Figure 7b) as evidence of their interactions with the surroundings using PCL, 0.5, and 2 wt% G scaffolds. For 1 and 3 wt% G, the cells had a round shape with protruding filaments indicating that they were entering the mitotic process. On days 7 and 14 (Figure 7c,d), almost all the cells had a round appearance, except for those on the PCL scaffold, which retained their flat shape, and the 3 wt% G scaffolds with a triangular appearance on day 7, 14, and 21 (Figure 7c–e). This indicates that the addition of G to the PCL scaffold enhanced both the proliferation and differentiation of cells [64,65].

SEM images show the adhesion, proliferation, and differentiation processes. The next step after cells adhere is proliferation, which is known as a mitotic process and requires the precise coordination of multiple signaling pathways [66]. It is affected by cell surface tension, intracellular pressure, and cortical stiffness. In the beginning, cells lose their capacity to adhere, and changes in intracellular pressure drive mitotic cells, thereby enabling them to exert a force against their surroundings. In previous studies, there was a correlation between changes in cortical stiffness and tension, such as Young’s modulus between the interphase and mitotic stages to resist whole-cell deformation [31,66]. Variations at different cell cycle stages are dependent on the depolymerization of the actin–myosin cortex, a network of filaments and contractile elements. This occurs through the increase in internal osmotic pressure, while depolymerization of actin filaments completely depends on the mechanosensing of the scaffold, which was influenced by mechanical properties. For example, a triangular shape showed on 3 wt% G (Figure 7c–e), but it was absent in the others [67,68].

## 4. Conclusions

Scaffolds for bone tissue engineering must have optimal physical, chemical, morphological, mechanical, biodegradable, and biocompatible properties for bone regeneration. The PCL/graphene (G) scaffold used in this research has the above characteristics, so it is an excellent scaffold. Due to the addition of G, PCL changes from hydrophobic (PCL) to hydrophilic (PCL/G). Compared with low concentrations of PCL/G (0.5, 1, 1.5 wt%) and PCL, the PCL/G scaffolds with high G concentrations (2, 2.5, and 3 wt%) had greater porosity. Therefore, the scaffold used in this research is suitable for the adhesion and growth of osteoblasts, especially because the scaffold’s Young’s modulus of 3 wt% G is close to that of trabecular bone. In addition, the results of the biocompatibility, proliferation, and differentiation experiments showed that the PCL/G scaffold was non-toxic, except for PCL and 0.5 wt% G, because its cell viability was lower than 70% (which is the basic requirement for human beings). Further future studies need to explore the long-term toxicity of graphene-based materials as well as the mechanism of mechanotransduction and mechanosensing to fully understand their effect and application.

## Figures and Tables

**Table 1:** Ratio I_D_/I_G_ band and I_2D_/I_G_ of PCL/G scaffold biodegradation over four months.

Scaffold	Ratio I_D_/I_G_ (COUNTS)					Ratio I_2D_/I_G_ (COUNTS)				
	Initial (*p* < 0.05)	Biodegradation				Initial (*p* > 0.05)	Biodegradation			
		1 Month (*p* > 0.05)	2 Months (*p* > 0.05)	3 Months (*p* > 0.05)	4 Months		1 Month (*p* > 0.05)	2 Months (*p*> 0.05)	3 Months (*p* > 0.05)	4 Months (*p* > 0.05)
2 wt% G	0.16 ± 0.02	0.15 ± 0.03	0.11 ± 0.01	0.06 ± 0.03	-	0.42 ± 0.03	0.63 ± 0.12	0.88 ± 0.05	1.20 ± 0.37	2.43 ± 1.44
2.5 wt% G	0.29 ± 0.04	0.19 ± 0.04	0.19 ± 0.05	0.10 ± 0.04	-	0.49 ± 0.08	0.65 ± 0.14	0.81 ± 0.08	1.31 ± 0.34	1.25 ± 0.87
3 wt% G	0.32 ± 0.04	0.25 ± 0.05	0.19 ± 0.02	0.11 ± 0.04	-	0.72 ± 0.26	1.13 ± 0.51	0.76 ± 0.13	1.37 ± 0.29	5.16 ± 4.58

**Table 2:** X-ray diffraction intensity of 2θ = 21.36°, 2θ = 23.6°, and 2θ = 26.48° of PCL/G scaffold over 4 months.

Scaffold	Intensity of 2θ = 21.36° (a.u)					Intensity of 2θ = 23.6° (a.u)					Intensity of 2θ = 26.48° (a.u)				
	Initial	Biodegradation (Month)					Biodegradation (Month)					Biodegradation (Month)			
		1	2	3	4	Initial	1	2	3	4	Initial	1	2	3	4
PCL	1780	2383.3	1915	1740	2035	690	803.3	675	640	785					
0.5 wt% G	1880	1570	2015	2000	1835	675	636.7	633.1	760	645					
1 wt% G	1885	1820	1888	1585	2035	650	665	805	665	765					
1.5 wt% G	2050	2065	1389	1175	3005	645	790	550	455	1155	110				255
2 wt% G	1433.3	2018	1395	3070	1696	570	876	515	1079.1	610	120	140.5	113.9	265	100.5
2.5 wt% G	1516.7	2050	1794.3	2435.9	1448.2	583.3	715	645.9	850	595	176.7	155.4	137.3	189.9	160
3 wt% G	1535	2015	1495	2866.6	1170	585	709	497.1	1021	415	195	270	165	308.9	135

## Data Availability

The datasets presented in this article are available for research purposes upon request from the corresponding author.
